# Postbiotic metabolites produced by *Lactobacillus plantarum* strains exert selective cytotoxicity effects on cancer cells

**DOI:** 10.1186/s12906-019-2528-2

**Published:** 2019-06-03

**Authors:** Li-Oon Chuah, Hooi Ling Foo, Teck Chwen Loh, Noorjahan Banu Mohammed Alitheen, Swee Keong Yeap, Nur Elina Abdul Mutalib, Raha Abdul Rahim, Khatijah Yusoff

**Affiliations:** 1 Department of Bioprocess Technology, Faculty of Biotechnology and Biomolecular Sciences, Universiti Putra Malaysia, 43400 UPM Serdang, Selangor Malaysia; 2 Institute of Bioscience, Universiti Putra Malaysia, 43400 UPM Serdang, Selangor Malaysia; 3 Department of Animal Science, Faculty of Agriculture, Universiti Putra Malaysia, 43400 UPM Serdang, Selangor Malaysia; 4 Institute of Tropical Agriculture, Universiti Putra Malaysia, 43400 UPM Serdang, Selangor Malaysia; 5 Department of Cell and Molecular Biology, Faculty of Biotechnology and Biomolecular Sciences, Universiti Putra Malaysia, 43400 UPM Serdang, Selangor Malaysia; 6 China-ASEAN College of Marine Sciences, Xiamen University Malaysia, 43900 Sepang, Selangor Malaysia; 7 Department of Microbiology, Faculty of Biotechnology and Biomolecular Sciences, 43400 UPM Serdang, Selangor Malaysia

**Keywords:** *Lactobacillus plantarum*, Postbiotic metabolites, Cytotoxicity, Antiproliferation, Haemolysis, Apoptosis

## Abstract

**Background:**

*Lactobacillus plantarum*, a major species of Lactic Acid Bacteria (LAB), are capable of producing postbiotic metabolites (PM) with prominent probiotic effects that have been documented extensively for rats, poultry and pigs. Despite the emerging evidence of anticancer properties of LAB, very limited information is available on cytotoxic and antiproliferative activity of PM produced by *L. plantarum*. Therefore, the cytotoxicity of PM produced by six strains of *L. plantarum* on various cancer and normal cells are yet to be evaluated.

**Methods:**

Postbiotic metabolites (PM) produced by six strains of *L. plantarum* were determined for their antiproliferative and cytotoxic effects on normal human primary cells, breast, colorectal, cervical, liver and leukemia cancer cell lines via MTT assay, trypan blue exclusion method and BrdU assay. The toxicity of PM was determined for human and various animal red blood cells via haemolytic assay. The cytotoxicity mode was subsequently determined for selected UL4 PM on MCF-7 cells due to its pronounced cytotoxic effect by fluorescent microscopic observation using AO/PI dye reagents and flow cytometric analyses.

**Results:**

UL4 PM exhibited the lowest IC_50_ value on MCF-7, RG14 PM on HT29 and RG11 and RI11 PM on HL60 cell lines, respectively from MTT assay. Moreover, all tested PM did not cause haemolysis of human, dog, rabbit and chicken red blood cells and demonstrated no cytotoxicity on normal breast MCF-10A cells and primary cultured cells including human peripheral blood mononuclear cells, mice splenocytes and thymocytes. Antiproliferation of MCF-7 and HT-29 cells was potently induced by UL4 and RG 14 PM respectively after 72 h of incubation at the concentration of 30% (v/v). Fluorescent microscopic observation and flow cytometric analyses showed that the pronounced cytotoxic effect of UL4 PM on MCF-7 cells was mediated through apoptosis.

**Conclusion:**

In conclusion**,** PM produced by the six strains of *L. plantarum* exhibited selective cytotoxic via antiproliferative effect and induction of apoptosis against malignant cancer cells in a strain-specific and cancer cell type-specific manner whilst sparing the normal cells. This reveals the vast potentials of PM from *L. plantarum* as functional supplement and as an adjunctive treatment for cancer.

## Background

Lactic acid bacteria (LAB) are generally accepted as safe, active and functional ingredients for foods owing to their long history of consumption together with fermented foods [1]. Furthermore, their metabolic end products, such as lactic acid and bacteriocin, can be used as natural preservatives and antimicrobial agents against contamination and food spoilage [2]. The beneficial effects of LAB have been reported extensively, such as the prevention of urogenital infections [3], control of inflammatory bowel diseases [4], immunomodulating functions [5, 6], control of serum cholesterol [7] and prevent certain types of cancer [8]. In addition, numerous in vivo, in vitro, human and epidemiological studies have provided increasing evidence of LAB effects on colon, bladder, liver, breast, and gastric cancers [8–12]. These effects are mediated by various mechanisms, such as the alteration of gastrointestinal microflora, enhancement of the host’s immune response, antioxidative, antiproliferative, and induction of apoptosis [13, 14]. Nevertheless, various LAB species employed as starter cultures in food fermentation may act as a reservoir for antibiotic resistance genes with the potential to be transferred to humans, animals and pathogenic microbes via food chain [15–19]. Antibiotic resistance is a major safety concern when the resistance is transferrable, especially to pathogenic bacteria [20]. Another concern for the application of living probiotic cultures is their viability below expected standards. This may affect their efficacy as viable commercial probiotic strains [21]. In addition, the functionality of probiotic cultures might be affected without changes in cell viability [22]. Several clinical studies demonstrated the use of live bacteria might possess adverse effects such as acute inflammation and increased mortality [23, 24].

Recently, there is growing interest in probiotic effects exerted by microbial metabolites known as bioactive postbiotic metabolites (PM), particularly in maintaining gut health, curing gut diseases and enhancing innate immunity [25–29]. PM ranges from soluble or secreted factors, metabolites, bacteriocins, and cell-free supernatant was reported with equivalent efficacy as live probiotics vastly related to Lactobacilli [24, 27]. Konstantinov et al. [28] summarised the role of postbiotics in maintaining colonic health and proposed that postbiotics can be a safer alternative in comparison to live bacteria. They further suggested the possibility of postbiotics in improving the patients’ quality of life in the later stage of colorectal cancer, while Tsilingiri et al. [24] proposed the utilisation of postbiotic in the treatment and prevention of gut-related diseases such as inflammatory bowel disease. Prominent probiotic effects of bacteriocin-containing postbiotic produced by the six strains of *Lactobacillus plantarum* employed in this study have been documented extensively for rats [29–31], poultry [32, 33] and pigs [34] with growth- and health-promoting effects observed with the supplementation of postbiotics as antibiotic replacer in their feeds. These *L. plantarum* strains produce several PM such as lactic acid, acetic acid and bacteriocin [35, 36]. Interestingly, Moghadam et al. [37] and Tai et al. [38] reported *L. plantarum* I-UL4 harbours a novel combination of two bacteriocin genes, the plantaricin W and plantaricin EF with broad inhibition activity against pathogenic Gram-positive and Gram-negative bacteria [35, 36].

The effects of different components (culture supernatants, cytoplasmic extracts, cell-wall extracts and live cells) of *Lactobacillus gasseri* and *Lactobacillus crispatus* on proliferative and apoptotic responses of normal and tumour cervical cells have been reported by Motevaseli et al. [39]. In addition, the exopolysaccharide from *L. plantarum* 70,180 have been reported to exert antitumor activity against colon carcinoma cells [40]. The culture supernatants of *Lactobacillus casei* and *Lactobacillus paracasei* strains isolated from human breast milk that have bioactivity of cytotoxicity and apoptosis against cervical cancer cells were suggested to have the potential as natural antitumour drugs [41]. Therefore, the cytotoxicity of PM produced by six strains of *L. plantarum* on various cancer and normal cells was determined in this study. In addition, the toxicity of the selected PM on human, dog, chicken and rabbit red blood cells (RBC) was subsequently verified. Cancer is a global epidemic disease affecting people at all ages and socio-economic groups. According to the report of GLOBOCAN (2012) [42], breast cancer incidence, mortality and 5-year (2012–2016) prevalence was estimated to be the highest for women, whereas colorectal cancer incidence, mortality and 5-year (2012–2016) prevalence were ranked second and third for women and men, respectively. Hence, the cytotoxic effect of PM produced by six strains of *L. plantarum* was further investigated on breast and colon cancer cell lines. The mode of cell death induced by the selected PM produced by *L. plantarum* I-UL4 was subsequently verified on breast cancer cells.

## Materials and methods

### Bacterial cultures

Six bacteriocin-producing *L. plantarum* identified as I-UL4, TL1, RS5, RI11, RG11 and RG14 strains [43] were isolated from Malaysian foods [44] and obtained from the Laboratory of Industrial Biotechnology, Department of Bioprocess Technology, Faculty of Biotechnology and Biomolecular Sciences, Universiti Putra Malaysia (UPM). They were maintained in De Man, Rogosa and Sharpe (MRS) medium (Merck, Germany) and propagated twice in MRS broth for 24 h at 30 °C under anaerobic condition prior to each experiment. The stock cultures were kept at -80 °C in MRS broth containing 20% (v/v) sterile glycerol.

### Cell culture and maintenance

Human breast cancer cells MCF-7 (ATCC-HTB-22), colorectal cancer cells HT-29 (ATCC HTB-38), cervical cancer cells HeLa (ATCC CCL2), liver cancer cells Hep-G2 (ATCC HB-8065), leukemia cells HL60 (ATCC CCL-240) and K562 (ATCC CCL-243) were provided by Animal Tissue Culture Laboratory of UPM. The nonmalignant MCF-10A cells (reference of normal glandular epithelium) were purchased from American Type Culture Collection (ATCC). All cells were maintained at 37 °C under 5% CO_2_ atmosphere using ATCC recommended medium supplemented with 10% (v/v) heat-inactivated fetal bovine serum and 100 IU/ml penicillin-streptomycin.

### Preparation of normal mice splenocytes and thymocytes, human peripheral blood mononuclear cells

The protocols of animal and human cell experiments were conducted according to the guidelines of the Institutional Animal Care and Use Committee (IACUC) of UPM. The 7–8 week old male mice were sacrificed by cervical dislocation. A transverse cut was made in the middle of the abdominal area to remove the spleen. The rib cage was cut off to remove the thymus. The spleen and thymus were then smashed separately by using wire mesh in phosphate buffer saline (PBS) and centrifuged at 200×*g* for 10 min. The cell pellet of splenocyte was re-suspended in 4 ml of lysis buffer and left on ice for 5 min prior to centrifugation at 200×*g* for 10 min. The splenocyte was then washed with 4 ml of PBS. For the thymocytes, the cell pellet was washed with PBS twice.

Human peripheral blood mononuclear cells and blood samples were withdrawn from healthy donors and kept in preservative-free heparin tubes (BD Biosciences, USA). Anticoagulated blood was diluted with an equal volume of PBS (pH 7.5) and slowly layered over Ficoll-Paque Plus (GE Healthcare, Sweden), followed by centrifugation at 400×*g* for 40 min at 18–20 °C. Plasma, mononuclear, Ficoll-Paque Plus, granulocytes and erythrocytes were separated into four layers. The mononuclear layer was transferred to a centrifuge tube and washed with 4 ml of PBS (pH 7.5) twice after the plasma layer was aspirated off.

The washed cell pellets of mice splenocytes, thymocytes and human peripheral blood mononuclear cells (PBMC) were re-suspended in complete growth media to a density of 5 × 10^5^ cells ml^− 1^ for MTT assay.

### Preparation of PM

Respective *L. plantarum* [1% (v/v)] was grown in MRS broth for 24 h at 30 °C. The cells were then separated by centrifugation at 10,000×*g* for 10 min at 4 °C. The supernatant was collected as PM. The pH of each PM was then adjusted to physiological pH (pH 7.2–7.4) using 5 M sodium hydroxide and all tested PM were then filtered through 0.22 μm polyethersulfone membrane syringe filter (Millipore, USA) prior to cytotoxicity, haemolysis, antiproliferation, and cell dead mode assays.

### Cytotoxic effect of PM on various cancer and normal cells

Cancer cells were plated onto 96-well microplates at 1 × 10^5^ cells ml^− 1^ and incubated at 37 °C in a 5% CO_2_ incubator for cytotoxicity assay. After 24 h, two-fold dilution of PM produced by *L. plantarum* strains were administered in complete growth medium with concentrations ranging from 0.47–30% (v/v). After each respective incubation interval (24, 48 and 72 h), 20 μl of 3-(4,5-dimethylthiazol-2-yl)-2,5-diphenyl tetrazolium bromide (MTT) solution (Sigma, USA) (5 mg ml^− 1^ in PBS) were added to each well and the plates were incubated in the dark for 4 h prior to centrifugation at 200×*g* for 5 min to separate the formazan crystals. The resultant formazan crystals were dissolved in 100 μl of dimethylsulfoxide (Fisher Scientific, UK) for 15–30 min after removing 170 μl of the growth medium from each well. The absorbance of formazan dye was quantified by a μ Quant ELISA reader (Biotek EL340, USA) at 570 nm with a reference wavelength of 630 nm. The experiment was repeated three times with triplicate samples. The following equation was used to calculate the percentage of cell viability: *(A*_*sample*_*-A*_*blank*_*)/(A*_*control*_*-A*_*blank*_*) × 100%*, where *A*_*sample*_ represents the absorbance of cells treated with PM; *A*_*blank*_ represents absorbance of PM and *A*_*control*_ represents absorbance of untreated cells. The concentration for 50% of growth (IC_50_) was determined by plotting the percentage of cell viability versus the concentration of PM.

### Haemolytic effect of PM on red blood cells

Haemolysis of red blood cell (RBC) was determined as described by Reddy et al. [45] with minor modifications. The fresh animal blood samples were obtained directly from the slaughter house of UPM. The collection of human peripheral blood mononuclear cells and blood samples were also approved by the IACUC of UPM. Human blood samples were withdrawn from healthy donors after consent was informed according to the protocol of IACUC of UPM. Washed RBC [2% (v/v)] were incubated with 100 μL of two-fold increasing concentrations [1.56–100% (v/v)] of PM prepared by using PBS in 96-round bottom well plates for 10 min.

The intact RBC were then separated by centrifugation at 400×*g* for 5 min and the absorbance of the supernatant was determined at 575 nm with a reference wavelength at 540 nm. Percentage of haemolysis was calculated by using the following formula: *(A*_*sample*_*-A*_*control*_*-A*_*blank*_*)/(A*_*100% lysis*_*-A*_*control*_*-A*_*blank*_*) × 100%.* Zero and 100% haemolysis were determined with isotonic PBS and deionised water. Two-fold diluted PM in PBS without RBC was used as blank, while 1% (v/v) sodium dodecyl sulfate (SDS) was served as positive control. The experiments were repeated three times with triplicate samples. The concentration of PM resulting in 50% lysis (L_50_) was determined from dose-response curve.

### Bromodeoxyuridine cell proliferation assay

Bromodeoxyuridine (BrdU) cell proliferation assay was conducted to study the antiproliferation effect of PM on breast and colon cancer cells. A cell population of 5 × 10^3^ cells well^− 1^ was seeded in a 96-well plate. Two controls were prepared: treatment blank (without cells) and background control (containing cells without BrdU reagent). The growth medium was aspirated after 24 h and replaced with fresh medium containing 0, 15 and 30% (v/v) PM and incubated for 24, 48 and 72 h, respectively. Further procedure was carried out according to the protocol of manufacturer (Millipore). The colored reaction product was quantified by measuring the absorbance at 450 nm (reference wavelength 540 nm) using a microplate reader. Three independent experiments were performed with triplicate samples. The following formula was used to calculate the percentage of cell proliferation: *(A*_*sample*_*-A*_*blank*_*)/(A*_*control*_*-A*_*blank*_*) × 100%*. The percentage of antiproliferation was obtained by subtracting 100 with the percentage of cell proliferation.

### Trypan blue exclusion assay

Cells were plated in 6-well tissue culture plate (2.5 × 10^5^ cells well^− 1^) and incubated for 24 h. The medium was then aspirated and replenished with fresh medium containing 0, 15 and 30% (v/v) PM. The cells were trypsinised at 0, 24, 48 and 72 h of incubations and collected by centrifugation at 200×*g* for 5 min. The supernatant was aspirated and the cell pellet was re-suspended in 100 μl of complete growth media.

Cell counting was performed by using hemocytometer. Ten microliter of cells was added with 10 μl of trypan blue dye and viewed under inverted microscope. The viable cells that did not stain blue were counted. The experiment was repeated three times with triplicate samples. Results were expressed as cell population, where it equals to the viable cell concentration in a single well multiplied with 100 μl of complete media that used to re-suspend the cell pellet.

### Fluorescent microscopy using acridine orange/propidium iodide staining

MCF-7 cells (2 × 10^5^ cells well^− 1^) seeded in six well plates were treated with 0% (v/v), 15% (v/v) and 30% (v/v) of PM and incubated in 5% CO_2_ atmospheric condition at 37 °C for 24, 48 and 72 h. After each incubation time, the detached cells in the medium were collected and mixed with trypsinised cells. The cells were then washed with PBS and incubated for 10 min with 10 μl of AO (100 μg ml^− 1^) and PI (100 μg ml^− 1^) at a ratio of 1:1 in 1 ml of cells. The stained cells were collected by centrifugation at 200×*g* for 5 min. The supernatant was aspirated and left 50 μl of remnant supernatant. The cell pellet was re-suspended and 10 μl of cell suspension was placed on slide and viewed under fluorescent microscope (Nikon FC-35DX, Japan) within 30 min using an excitation filter and barrier filter at 450–490 nm and long pass filter of 520 nm. Viable (green intact cells), apoptotic (green shrinking cells with condensed or fragmented nucleus), late apoptotic and necrotic (red) cells were observed. The experiment was repeated for at least three times with triplicate samples.

### Flow cytometry analysis

Cell cycle analysis and Annexin V was performed to determine the cell cycle perturbation of MCF-7 cells when treated with postbiotic UL4 at different concentrations [15 and 30% (v/v)] and different incubation periods (24, 48 and 72 h). Phosphatidylserine exposure on the cell surface of MCF-7 cells treated with postbiotic UL4 was detected using Annexin V-FITC Apoptosis Detection Kit (BD Bioscience, USA) according to manufacturer’s protocol.

### Statistical analysis

Data of cytotoxicity and antiproliferative effects were analysed by using ANOVA with all paired-wise multiple comparison procedure (Tukey) and presented as the mean ± standard error of the mean (SEM). The results were analysed by Minitab Statistical Software at differences of *P* < 0.05.

## Results

### Cytotoxic effect of PM on various cancer and normal cells

The inhibition concentration of 50% growth (IC_50_) were determined for various cancer cell lines to compare the cytotoxicity effects of PM produced by *L. plantarum* I-UL4, TL1, RS5, RG14, RG11 and RI11, which was designated as UL4 PM, TL1 PM, RS5 PM, RG14 PM, RG11 PM and RI11 PM, respectively. The cytotoxic effect of the PM was time- and dose-dependent [41]. No cytotoxicity activity was noted for MRS medium against various cancer cell lines in this study (results not shown). Table 1 shows the PM produced by *L. plantarum* strains exhibited profound cytotoxic effect at 72 h of incubation on all tested cancer (breast, cervical, colorectal, liver and leukemia) cell lines, although different IC_50_ values were exhibited by different PM. Moreover, the tested PM exhibited more potent cytotoxic effect on suspension cancer cells such as HL60 and K562 as compared to anchorage-dependent cancer cells (such as MCF-7, HeLa, Hep-G2 and HT-29). This was particularly evident on HL60 in comparison to K562. Interestingly, amongst the anchorage-dependent cells, the PM produced by all *L. plantarum* strains was more cytotoxic against MCF-7 cells. Among them, UL4 PM exhibited the most potent cytotoxic effect against MCF-7 cells with IC_50_ of 10% (v/v) at 72 h of incubation (Table 1).

**Table 1:** IC_50_ values of PM produced by six strains of *Lactobacillus plantarum* on various cancer cells

	IC_50_ value of PM produced by *L. plantarum* strains, % (v/v)					
Cancer Cells	UL4	TL1	RS5	RG14	RG11	RI11
MCF-7 (ATCC®HTB-22)	10	13	21	20	16	16
HeLa (ATCC® CCL2)	20	18	24	20	N.D.	18
Hep-G2 (ATCC®HB-8065)	22	22	27	22	N.D.	N.D.
HT-29 (ATCC®HTB-38)	N.D.	N.D.	28	22	N.D.	N.D.
K562 (ATCC®CCL-240)	10	5	5	5	5	5
HL60 (ATCC®CCL-243)	5	5	9	10	1	1

Notes: Human breast cancer cells MCF-7 (ATCC-HTB-22), colorectal cancer cells HT-29 (ATCC HTB-38), cervical cancer cells HeLa (ATCC CCL2) and liver cancer cells Hep-G2 (ATCC HB-8065) are anchorage-dependent cells while leukemia cells HL60 (ATCC CCL-240) and K562 (ATCC CCL-243) are suspension cells. The highest concentration of PM being tested on all cells was 30% (v/v). The IC_50_ values were detected after 72 h of incubation. The values reported are the means of nine replicates. N.D., no IC50 values were detected up to the concentration of 30% (v/v)

Despite the profound cytotoxic effects of PM on various cancer cells, no IC_50_ value was detected on rapidly-dividing normal MCF-10A cells, with the exception that limited cytotoxicity [IC_50_ value of 26% (v/v)] was detected when treated with UL4 PM for 72 h (results not shown).

### Haemolytic effect of PM on red blood cells

**Fig. 1:**
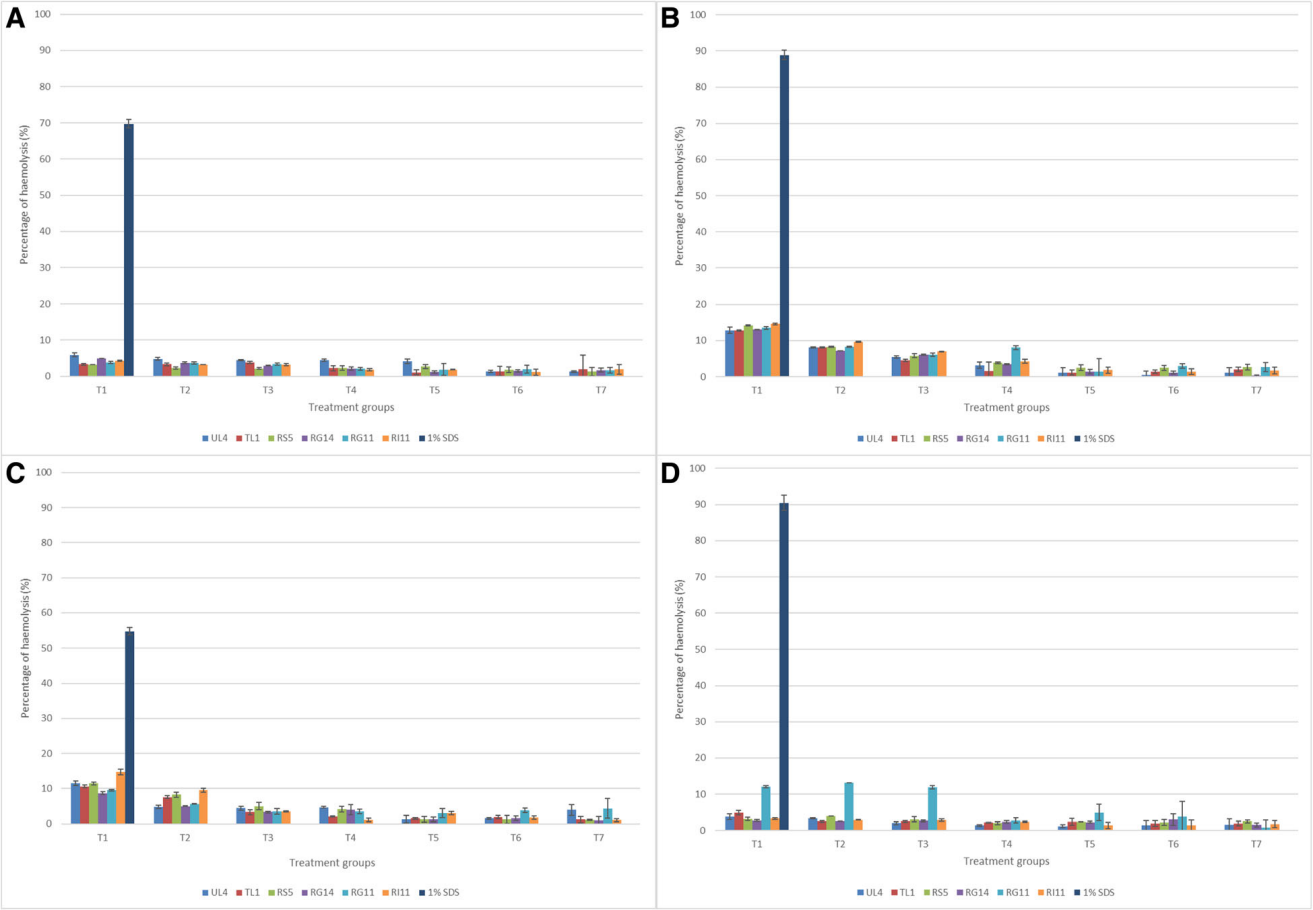
Haemolysis effect of PM produced by six stains of *Lactobacillus plantarum* on (**a**) human, (**b**) rabbit, (**c**) chicken and (**d**) dog RBC. T1: 100% (v/v); T2: 50% (v/v): T3: 25% (v/v); T4: 12.5% (v/v); T5: 6.25% (v/v); T6: 3.13% (v/v); T7: 1.56% (v/v); 1% SDS: 1% (w/v) SDS. Each bar represents the mean ± SEM of nine replicates

The haemolytic effect of PM produced by six strains of *L. plantarum* was determined with red blood cells (RBC) of humans, dogs, rabbits and chickens. With respect to lytic concentration at equilibrium (which was usually expressed as L_50_ concentration), toxicity on the basis of RBC haemolysis was not detected for all tested PM although the concentration was increased to 100% (v/v) as compared to 1% (v/v) SDS (Fig. 1).

### Antiproliferative activities of PM on breast and colon cancer cells

In this study, the antiproliferative activities evaluated by Bromodeoxyuridine(BrdU) proliferation assay of PM produced by *L. plantarum* I-UL4, TL1, RS5, RG14, RG11 and RI11 were particularly focused on the breast and colon cancer cells due to the high incidence and mortality of both cancers as reported by GLOBOCAN (2012) [42], in addition to extensive studies reported on LAB and their anticancer effects on colorectal cancer [8, 14].

**Fig. 2:**
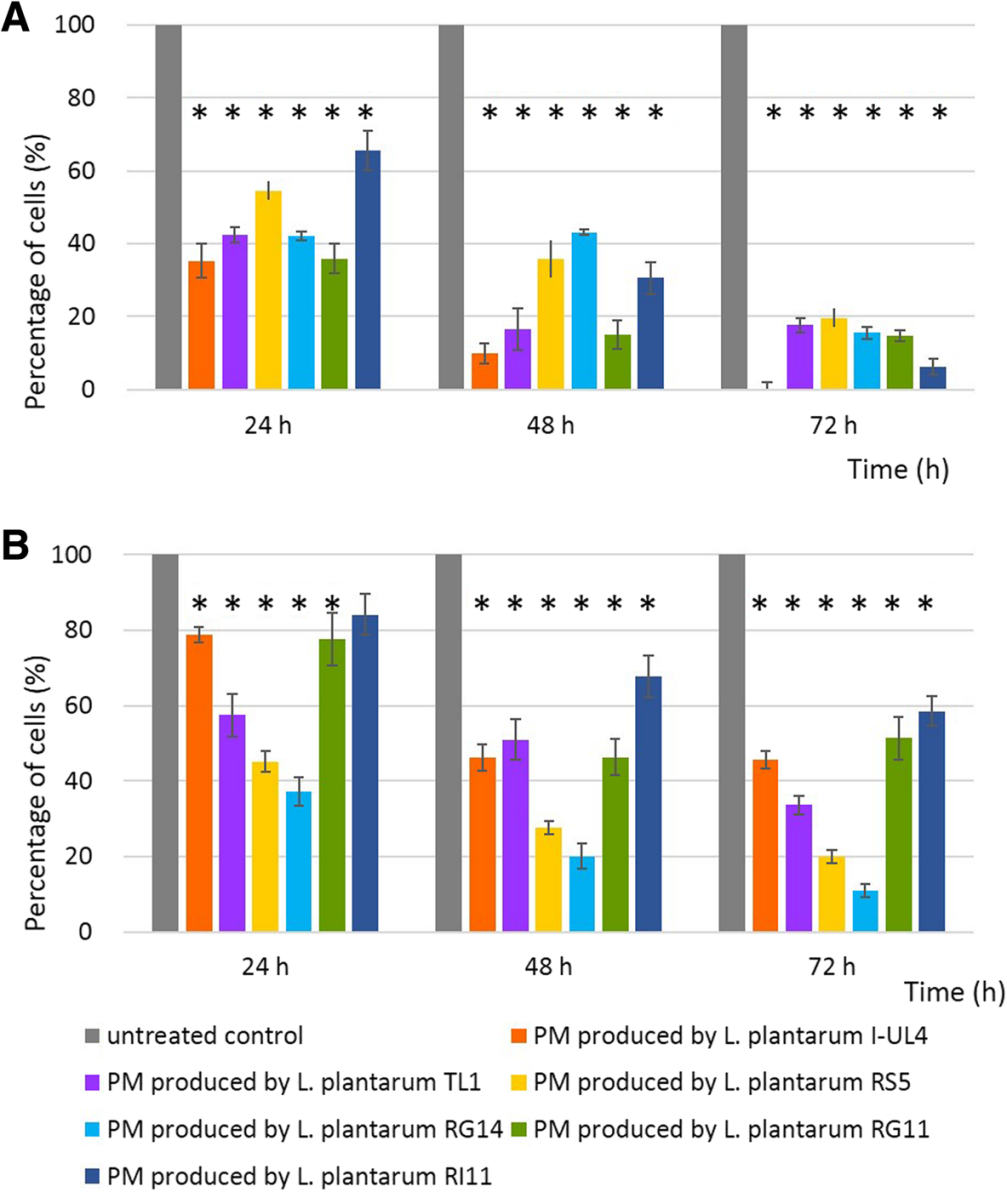
Antiproliferative effect of 30% (v/v) of PM produced by six strains of *Lactobacillus plantarum* on (**a**) MCF-7 cells and (**b**) HT-29 cells determined by BrdU cell proliferation assay. Values within the same row and experiment having an asterisk indicate significant differences (*P* < 0.05) from the untreated control groups. Error bars represent the standard error of the mean of nine replicates

Significant antiproliferative effect (*P < 0.05*) on MCF-7 (Fig. 2 panel a) and HT-29 (Fig. 2 panel b) were detected for all PM at the concentrations of 15% (v/v) (results not shown) and 30% (v/v), respectively, whereby continuous reduction of BrdU incorporation was noted with increased concentrations of PM and incubation time as shown in Fig. 2. In comparison, all tested PM decreased the rate of DNA synthesis in MCF-7 cells (Fig. 2 panel a) more profoundly than in HT-29 cells (Fig. 2 panel b) throughout the incubation times used in this study. At 72 h of incubation, 30% (v/v) concentration of PM produced by *L. plantarum* I-UL4 ceased the DNA synthesis of MCF-7 cells remarkably, whereas small amount of BrdU was incorporated into MCF-7 cells when treated with other PM at the concentration of 30% (v/v) (Fig. 2 panel a). As for HT-29 cells, RG14 PM demonstrated the highest reduction (89%) of DNA synthesis in HT-29 cells as compared to the PM produced by the other *L. plantarum* strains (Fig. 2 panel b).

**Fig. 3:**
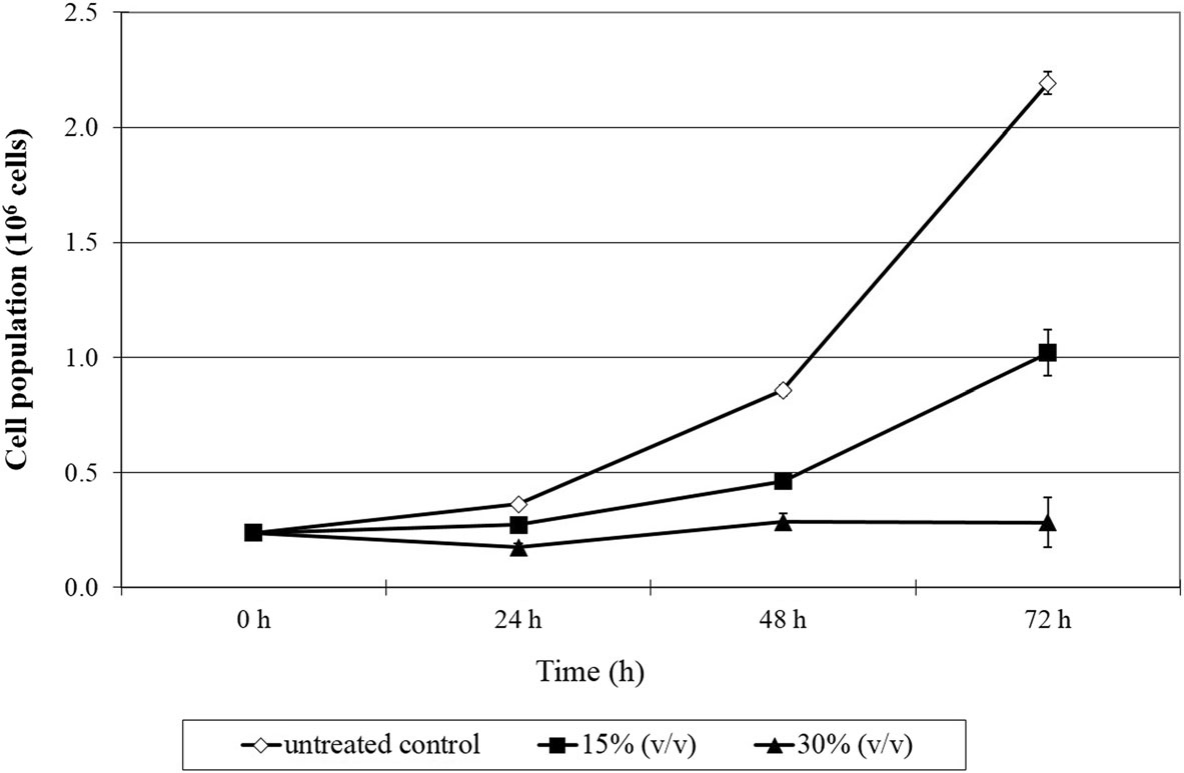
Antiproliferative effect of PM produced by *Lactobacillus plantarum* I-UL4 on MCF-7 cells determined via trypan blue staining and cell count. Cell population equals to the viable cell concentration of a single well multiplied with 100 μl of complete growth media used to suspend the cell pellet. Values within the same row and experiment having an asterisk indicate significant difference (*P* < 0.05) from the untreated control groups. Error bars represent the standard error of the mean

Further verification of antiproliferative effects of PM produced by *L. plantarum* was conducted for growth arrest study via trypan blue cell counting. PM produced by *L. plantarum* I-UL4 and RG14 with distinct antiproliferative property on MCF-7 cells and HT-29 cells respectively were selected and the viable cell count was enumerated. Figure 3 shows that PM produced by *L. plantarum* I-UL4 exhibited time and dose-dependent inhibition on proliferation of MCF-7 cells. Proliferation of MCF-7 cells was significantly arrested by UL4 PM at the concentration of 30% (v/v). Pronounced differences in cell population were observed among 15% (v/v) and 30% (v/v) PM-treated MCF-7 cells and the untreated cells. This difference became more significant (*P < 0.05*) with prolong incubation time. After 72 h of incubation, the ratio of viable population of MCF-7 cells incubated with 15% (v/v) and 30% (v/v) of UL4 PM to the viable population of untreated cells were 1:2 and 1:1.3 at 24 h; 1:3 and 1:1.9 at 48 h; and 1:7.7 and 1:2.2 at 72 h, respectively.

**Fig. 4:**
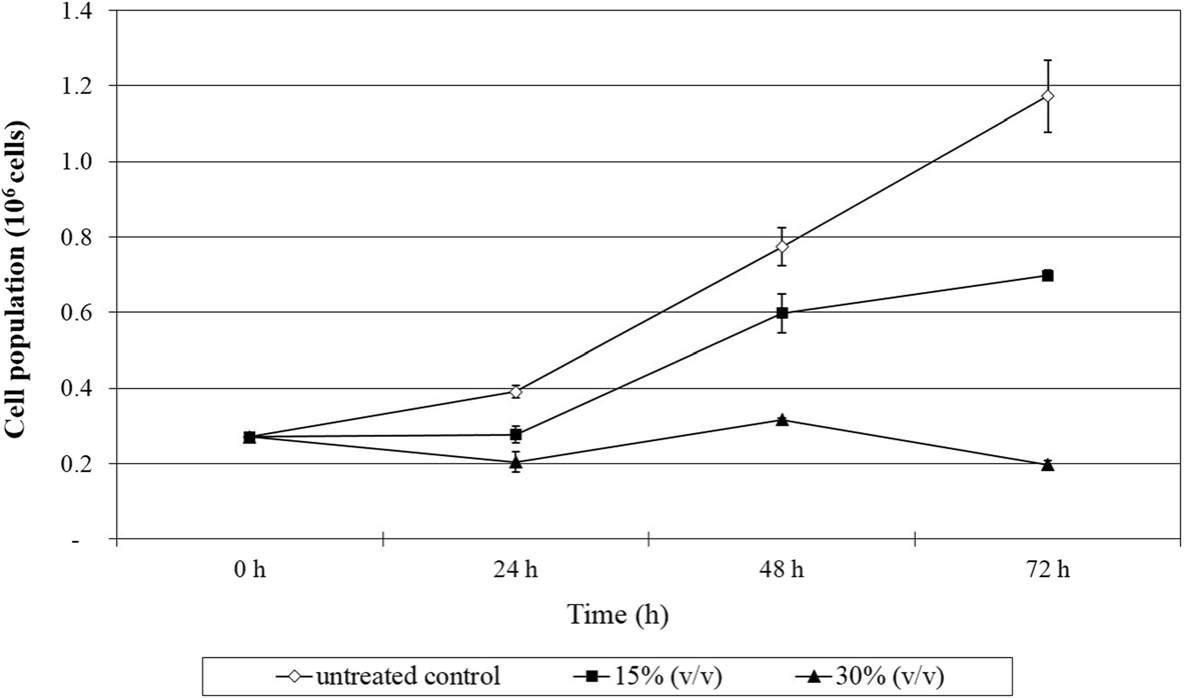
Antiproliferative effect of PM produced by *Lactobacillus plantarum* RG14 on HT-29 cells determined via trypan blue staining and cell count. Cell population equals to the viable cell concentration of a single well multiplied with 100 μl of complete growth media used to suspend the cell pellet. Values within the same row and experiment having an asterisk indicate significant difference (P < 0.05) from the untreated control groups. Error bars represent the standard error of the mean

As for HT-29 cells, significant difference in cell population was observed for HT-29 cells treated with 15% (v/v) and 30% (v/v) RG14 PM, as well as the untreated cells. RG14 PM exhibited time-and dose-dependent antiproliferative effects on HT-29 cells, whereby the ratio of viable population of HT-29 cells treated with 15% (v/v) and 30% (v/v) of RG14 PM to the viable population of untreated population were 1:1.6 and 1:1.3 at 24 h; 2.1 and 1:1.3 at 48 h; and 1:4.3 and 1:1.5 at 72 h, respectively (Fig. 4).

### Apoptosis induced by UL4 PM

**Fig. 5:**
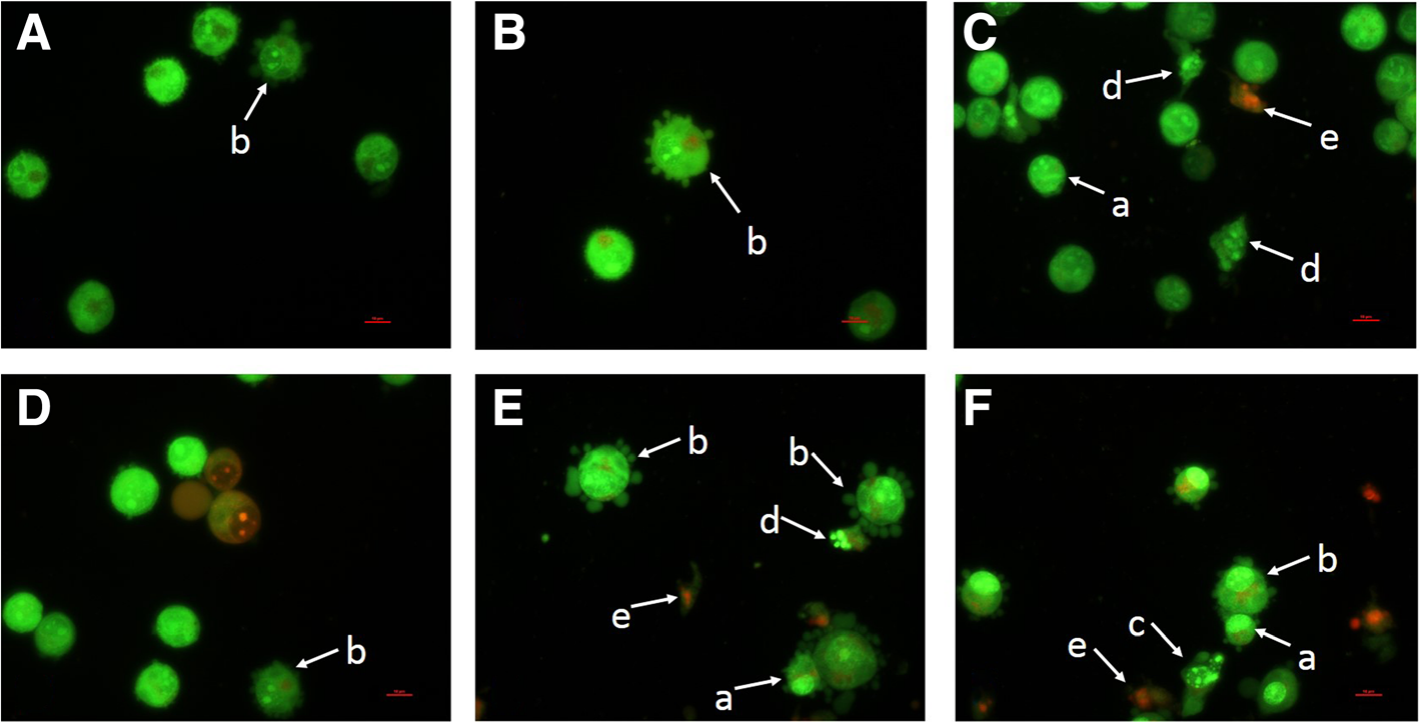
Apoptosis induction by PM of *Lactobacillus plantarum* I-UL4 on MCF-7 cells. Panel **a**: 15% (v/v), 24 h; **b**: 15% (v/v), 48 h; **c**: 15% (v/v), 72 h; **d**: 30% (v/v), 24 h; **e**: 30% (v/v), 48 h; and **f**: 30% (v/v), 72 h. Morphological changes following exposure to PM produced by *L. plantarum* I-UL4 were typical of apoptosis, i.e. cell shrinkage (a), membrane blebbing (b), perinuclear chromatin condensation (c), apoptotic bodies formation (d), and secondary necrotic cells (e). (Bar: 10 μm; magnification: 400×)

Results obtained in this time course study showed that 15 and 30% (v/v) UL4 PM induced apoptotic cell death in MCF-7 cells. Figure 5 shows different morphologies of MCF-7 cells when treated with UL4 PM as detected by using acridine orange (AO) and propidium iodide **(**PI) staining. Cell shrinkage, masses of condensed chromatin aggregation at the nuclear membrane as shown by bright fluorescence at the membrane periphery, membrane blebbing and cytoplasmic and nuclear fragmentation leading to the formation of apoptotic bodies were among the striking apoptotic cellular changes noted in the treated MCF-7 cells observed under fluorescence microscope.

Flow cytometric assessments via cell cycle perturbation and phospatidylserine exposure using Annexin V verified further the apoptotic effect of UL4 metabolites on MCF-7 cells. Cell cycle was predominantly arrested at G_0_/G_1_ phase after 48 h of incubation for both treatment groups of 15% (v/v) and 30% (v/v) (Table 2), while apoptotic cells were majorly predominating the cell population in a dose- and time-dependent manner especially when treated with 30% (v/v) UL4 PM (Table 3).

**Table 2:** Effect of postbiotic UL4 on cell cycle phases distribution of MCF-7 cells

	Untreated control (%)	15% (v/v) (%)	30% (v/v) (%)
24 h			
Sub- G_0_/G_1_	0.5^a^ ± 0.3	1.7^ab^ ± 1.6	1.2^a^ ± 1.0
G_0_/G_1_	59.1^ab^ ± 3.1	64.5^bd^ ± 6.7	74.2^b^ ± 9.5
S	21.4^a^ ± 4.2	16.4^ac^ ± 3.6	12.8^ac^ ± 5.7
M	19.3^a^ ± 1.6	17.6^ac^ ± 3.3	12.3^ac^ ± 3.5
48 h			
Sub- G_0_/G_1_	0.5^a^ ± 0.2	14.3^bc^ ± 5.6	15.5^cd^ ± 1.7
G_0_/G_1_	80.1^bc^ ± 6.2	62.7^be^ ± 2.1	56.3^be^ ± 4.9
S	9.4^ac^ ± 3.7	13.7^ac^ ± 2.7	13.3^ac^ ± 3.0
M	10.1^ac^ ± 2.6	9.7^ac^ ± 1.1	14.2^ac^ ± 3.6
72 h			
Sub- G_0_/G_1_	0.5^a^ ± 0.1	9.5^abc^ ± 4.7	27.5^d^ ± 1.2
G_0_/G_1_	89.9^c^ ± 1.4	70.2^be^ ± 3.4	48.0^ade^ ± 4.2
S	3.3^bc^ ± 0.4	9.9^ac^ ± 1.1	12.7^ac^ ± 1.2
M	6.2^bc^ ± 1.5	10.1^ac^ ± 1.2	11.6^ac^ ± 2.9

Notes: The data represent the mean of percentage of cells. Values shown represent mean ± SEM. Values within the same row and column sharing a common superscript letter are not significantly different (*P* > 0.05)

**Table 3:** Cell death modes of MCF-7 cells induced by postbiotic UL4

	Untreated control (%)	15% (v/v) (%)	30% (v/v) (%)
24 h			
Viable cells	86.7^a^ ± 1.5	84.8^a^ ± 1.3	52.2^b^ ± 6.3
Apoptotic cells	11.9^a^ ± 1.4	13.9^ab^ ± 2.0	30.1^ab^ ± 4.2
Necrotic cells	1.4^a^ ± 1.1	1.4^a^ ± 0.7	17.7^bc^ ± 2.2
48 h			
Viable cells	92.2^a^ ± 1.2	79.1^a^ ± 0.6	24.7^d^ ± 7.2
Apoptotic cells	6.5^a^ ± 1.5	17.3^a^ ± 0.5	65.9^c^ ± 9.8
Necrotic cells	1.3^a^ ± 0.7	3.7^ab^ ± 0.1	9.4^ac^ ± 2.6
72 h			
Viable cells	88.1^a^ ± 1.1	48.6^c^ ± 0.1	14.7^d^ ± 3.5
Apoptotic cells	9.6^a^ ± 0.6	38.8^b^ ± 1.2	60.0^c^ ± 4.3
Necrotic cells	2.3^a^ ± 0.5	12.7^abc^ ± 1.3	25.3^c^ ± 7.7

Notes: Percentages of viable, early apoptotic, and late apoptotic and necrotic cells were scored based on their morphological features. The data represent the mean of percentage of cells. Values shown represent mean ± SEM. Values within the same row and column of a particular cell type sharing a common superscript letter are not significantly different (*P* > 0.05). The cell death mode of MCF-7 cells was analyzed by fluorescent microscopy

## Discussion

Food has long been known to be a vital source to maintain well-being and health status. Nowadays, the functional food market is in the limelight and gains more attention from consumer on the functionality of foods. However, some of these foods are costly and the unclear safety of the food components becomes drawbacks to consumers [46]. Postbiotic containing bacteriocins, for example, are constituted with small peptides, which are mostly non-immunogenic and biodegradable, thus safe for consumption and would not contaminate the environment when disposed [47]. Furthermore, the utilisation of postbiotic are not limited to functional foods, probiotic-mediated suppression of cancer or inflamed cells have also been reported for postbiotic [10, 24, 25]. Postbiotic from lactobacilli offers multitude possibilities for utilisation in cancer cells and inflammatory studies [48]. This offers a safer alternative to the current treatment of radio- and chemotherapies that is limited by toxicities associated with the latter therapies. In this present study, we investigated the cytotoxic effect of PM produced by six strains of *L. plantarum* on cancer and normal cell line to determine the potential of the PM as a functional supplement and as an adjunctive treatment for cancer.

As shown in this study, RG11 PM and RI11 PM exhibited remarkable cytotoxic effect on all tested suspension cancer cells (K562 and HL60) but they did not exhibit any significant toxicity on epithelial-origin cancer cells (HeLa, Hep-G2 and HT-29) except for MCF-7 breast cancer cells. In addition, only RG14 PM and RS5 PM showed cytotoxic effects on HT-29 cells with IC_50_ values of 22 and 28% (v/v), respectively. The strain-specific and cell type-dependent cytotoxic effects of PM were in agreement with the finding of Chumchalova and Smarda, whereby the inhibitory effects of colicins were cell-, species- and strain-specific and act in dose-dependent manner [49]. Furthermore, a study conducted by Tan et al. [50] demonstrated that different media compositions of reconstituted MRS with the supplementation of polysorbate 80 affected significantly the selective cytotoxic and antiproliferative effects of PM produced by *L. plantarum* I-UL4 on MCF-7 cells.

Assessment of potential risk to human health is essential for the products including those for food, home care, personal care, pharmaceutical use and pesticides [51]. Most cytotoxic agents are not only targeting to the rapidly dividing cancer cells, but also simultaneously giving deleterious side effect to some rapidly dividing normal cells. Some normal tissues are also sensitive to the induction of cell death by cytotoxic agents [52, 53]. In this study, no marked toxicity on normal breast MCF-10A cells was noted, which agreed with Motasevali et al. [39] that have reported that culture supernatants of *L. gasseri* and *L. crispatus* are non-toxic to normal cervical cells. Furthermore, inert towards actively proliferating primary mice splenocyte, thymocytes and human peripheral mononuclear cells were detected for the tested PM, indicating their selectivity on malignant cells.

Other than normal cell line and primary cultured cells, erythrocytes or RBC are employed as a prime candidate for the determination of membranolytic or cytolytic activities. The PM produced by *L. plantarum* I-UL4 was employed for the haemolysis study since it exhibited profound cytotoxic effects on all tested suspension cancer cells and epithelial-origin cancer cells, except for HT-29 cells (Table 1). The results of haemolytic effect showed that there was no haemolysis observed for all RBC treated with UL4 PM, which agreed with the findings of Thirabunyanon et al. [54]. The haemolytic results implied that UL4 PM has no adverse effect on membrane permeability properties and the cytotoxic effect of PM observed in this study was not attributed to the osmotic pressure and acute toxicity effect on the treated cancer cells.

In addition, a myriad of health effects has been associated vastly to LAB and most of the studies on anticancer effects of LAB were demonstrated extensively on colorectal cancer [55]. Thus, MCF-7 and HT-29 cells were selected subsequently on the basis of their reported prevalence on the global population, for the determination of antiproliferation effect of PM. The results of the antiproliferative effect correlated well with cytotoxic effect of tested PM, which was also strain-specific and cell type-dependent. UL4 PM manifested the most profound antiproliferative effect on MCF-7 cells, whereas RG14 PM showed the most significant (*P < 0.05*) antiproliferative effect on HT-29 cells. In comparison, the antiproliferative effect on MCF-7 cells induced by UL4 PM was more profound that the RG14 PM on HT-29 cells. Nonetheless, the antiproliferative effects of UL4 PM on MCF-7 cells and RG14 PM on HT-29 cells were further verified by trypan blue assay and similar results were also obtained as shown in BrdU cell proliferation assay.

The mode of cell death induced by UL4 PM against MCF-7 cells was subsequently determined in this study since the UL4 PM exhibited the most potent cytotoxic effect on MCF-7 cells in comparison to cytotoxic effect manifested on HT-29 cells as well as the effect induced by the RG14 PM. Staining with fluorescence dyes such as AO and PI is considered as an appropriate method for evaluating the changes of nuclear morphology [56] associated with cytotoxic activity, which can be used to facilitate the differentiation of cell death mode, as well as the characteristic morphological changes in chromatin and cell membrane involved in cytotoxic activity induced by cytotoxic agent, as well as the detection of the cell cycle perturbation via flow cytometric analyses. Based on nuclear morphology (perinuclear chromatin condensation, nuclear collapse and eventually fragmentation), several subpopulations of apoptotic cells could be distinguished from viable and necrotic cells [57]. Morphological changes in a transition from membrane blebbing and nucleus condensation, cell shrinkage and apoptotic body formation to secondary necrotic bodies (which nucleus was stained orange-red but the cytoplasm was stained green) as observed in this study inferring that apoptosis rather than necrosis was induced by the UL4 PM. The cell cycle arrest at G_0_/G_1_ phase as well as detection of exposed phosphatidylserine on the cell surface via flow cytometric analyses supported further the notion of the apoptotic cell death mode of MCF-7 cells exerted by postbiotic UL4.

It is noteworthy that PM produced by the six strains of *L. plantarum* were selective on various human cancer cells, implying the vast potential of the PM as an alternative nutraceutical supplements that possess promising anticancer activity due to their low cytotoxicity to normal mammalian cells. Notably, PM produced by *L. plantarum* I-UL4 specifically induced apoptosis, resulting in the breakdown of apoptosised cells into smaller apoptotic bodies, which are then reported to be cleared by phagocytic cells without evoking any inflammatory reaction in vivo [58], a preferable cell mode death as compared to necrosis [59, 60] for the treatment of cancers.

## Conclusion

In conclusion, the results obtained in present study indicate that PM produced by *L. plantarum* exhibited selective cytotoxic effect on various cancer cells in dose- and time-dependent manners without causing toxic effect or haemolysis on normal cells. Fluorescent microscopic observation and flow cytometric assessments showed that UL4 PM reduced viability of MCF-7 cells via antiproliferative effect and induction of apoptosis. The selectivity of the PM produced by *L. plantarum* strains to various tumorigenic cells inferring that they hold interesting anti-cancer properties and hence possesses vast potential as a functional supplement and as an adjuncctive treatment against cancer which can be further explored.

## Data Availability

The datasets used and/or analysed during this study are available from the corresponding author on reasonable request.

## Abbreviations

ANOVA: Analysis of variance
AO: Acridine orange
ATCC: American Type Culture Collection
BrdU: Bromodeoxyuridine
DNA: Deoxyribonucleic acid
ELISA: Enzyme-linked immunoabsorbent assay
h: Hours
IACUC: Institute of Animal Care and Use Committee
ICR: Institute of Cancer Research
LAB: Lactic acid bacteria
MRS: de Man Rogosa Sharpe
MTT: 3-(4,5-dimethylthiazol-2-yl)-2,5-diphenyl tetrazolium bromide
PBMC: Peripheral blood mononuclear cells
PBS: Phosphate buffer saline
PI: Propidium iodide
PM: Postbiotic metabolites
RBC: Red blood cells
UPM: Universiti Putra Malaysia
